# Changes in stress pathways as a possible mechanism of aerobic exercise training on brain health: a scoping review of existing studies

**DOI:** 10.3389/fphys.2023.1273981

**Published:** 2023-10-11

**Authors:** Cristina Molina-Hidalgo, Chelsea M. Stillman, Audrey M. Collins, Daniel Velazquez-Diaz, Hayley S. Ripperger, Jermon A. Drake, Peter J. Gianaros, Anna L. Marsland, Kirk I. Erickson

**Affiliations:** ^1^ AdventHealth Research Institute, Neuroscience Institute, Orlando, FL, United States; ^2^ Department of Psychology, University of Pittsburgh, Pittsburgh, PA, United States; ^3^ ExPhy Research Group, Department of Physical Education, University of Cadiz, Cadiz, Spain

**Keywords:** cortisol, salivary alpha-amylase, physical activity, HPA axis, ANS

## Abstract

Physical activity (PA) in the form of aerobic exercise (AE) preserves and improves neurocognitive function across the lifespan. However, a mechanistic understanding of the pathways by which aerobic exercise impacts brain health is still lacking, particularly with respect to stress-related pathways. One mechanistic hypothesis is that AE improves neurocognitive health in part by modifying circulating levels of stress-related hormones and signaling factors associated with the hypothalamic-pituitary-adrenal (HPA) axis and autonomic nervous system (ANS), as commonly measured by the biomarkers cortisol (CORT) and salivary α-amylase (sAA). Thus, this hypothesis predicts that changes in stress biomarkers, such as CORT and sAA, are possible explanatory pathways mediating the positive effects of AE on neurocognitive health. In the present review article, we provide a summary of available studies examining the possibility that exercise-induced changes to stress biomarkers could partly account for exercise-related improvements in neurocognitive health. Our review indicates that despite the intuitive appeal of this hypothesis, there is insufficient evidence available to conclude that chronic and habitual AE affects neurocognitive health by altering stress biomarker pathways. The cross-sectional nature of the majority of reviewed studies highlights the need for well-controlled studies to adequately test this hypothesis.

## 1 Introduction

**FIGURE 1:**
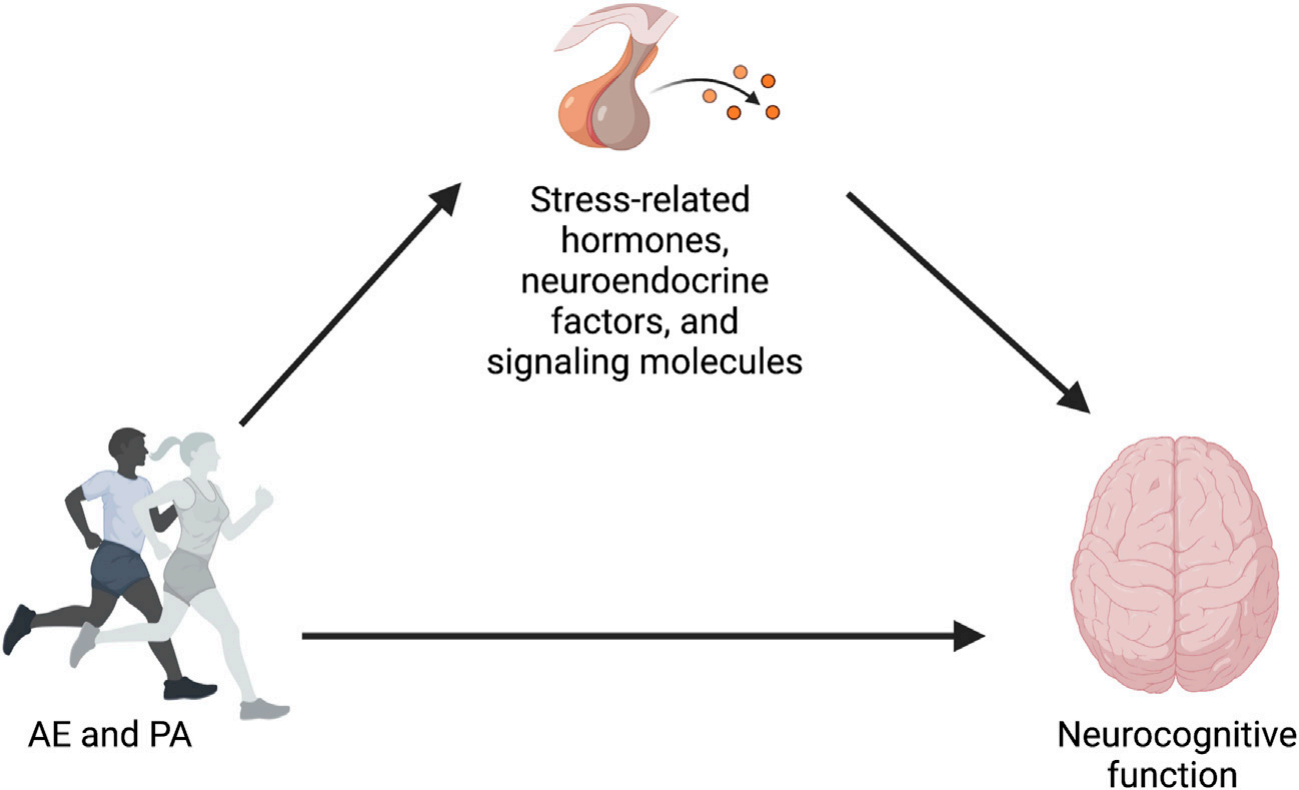
Conceptual model proposing changes in stress-related hormones, neuroendocrine factors, and signaling molecules as a possible mechanism explaining the positive effects of AE and PA on neurocognitive function.

Physical activity (PA), in the form of aerobic exercise (AE), is an accessible and cost-effective non-pharmaceutical approach to preserve and improve neurocognitive health across the lifespan (Erickson and Liu-Ambrose, 2016; Stillman et al., 2016; Erickson et al., 2019; Stillman et al., 2020). Unfortunately, there is still a poor understanding of the mechanisms by which PA and AE impact brain health and cognition in humans (Hillmanet al., 2008; Gomez-Pinilla and Hillman, 2013; Stillman et al., 2016; Chen et al., 2017; Stillman and Erickson, 2018; Stillman et al., 2020; Erickson et al., 2022; Chen and Nakagawa, 2023). While various pathways have been previously discussed, spanning from molecular and cellular to brain systems and behavioral mechanisms, there are other under-explored and less frequently discussed pathways that could play a role in this relationship. For example, one pathway that has not been explored in-depth, but warrants deeper exploration, is linked to stress. Stress hormones play a major role in mediating both adaptive and maladaptive responses, and they do so by interacting with specific aspects of the physiology of affected tissues. Prolonged exposure to stress may negatively impact health outcomes, including neurocognitive function (Frodl and O'Keane, 2013; Juster et al., 2010; Lupien et al., 2007). From this perspective, changes in stress-related hormones, neuroendocrine factors, and signaling molecules, as reflected by levels of their distal biomarkers, particularly circulating, urinary, and salivary cortisol (CORT) and salivary α-amylase (sAA), have been proposed as a possible mechanism explaining the positive effects of AE on neurocognitive health (see Figure 1). The reasoning is that if engagement in AE affects stress physiology, and if stress physiology influences neurocognitive health, then AE-induced changes to stress biomarkers could be used to measure one pathway by which the neurocognitive benefits of AE are realized. Thus, changes in stress biomarkers, as the cross-stressor adaptation hypothesis suggests, might partially explain the neurocognitive health benefits of AE. Also, it has been shown that AE decreases stress reactivity (i.e., decreased blood pressure or subjective ratings of perceived stress), which suggest that exercise may play a protective role against allostatic load (Epel et al., 2018).

The purpose of this scoping review is to 1) summarize and synthesize the results of AE interventions measuring stress biomarkers such as CORT and sAA and 2) determine their relationship to neurocognitive changes associated with AE training. Before we discuss the evidence for stress biomarker changes due to AE in relation to neurocognitive improvements, we will first define the relationship between stress biomarkers, stress exposure, and neurocognitive function, as they will be used throughout the following sections.

## 2 Stress biomarkers: CORT and SAA

The concept of stress has been used across disciplines with different methodological approaches. A global definition of this concept comes from Cohen et al. (2016), who defined stress as “*a set of constructs representing stages in a process by which environmental demands that tax or exceed the adaptive capacity of an organism occasion psychological, behavioral, and biological responses that may place persons at risk for disease*” (Cohen et al., 2016). According to the biological framework, *stress* is defined as a state in which an organism’s *homeostasis* is threatened (real or perceived) by internal or external adverse forces (emotional or physical), termed *stressors* (Chrousos, 2009). In response to stressors, two neuroendocrine pathways are activated. The sympathetic nervous system (SNS) responds rapidly leading to the release of catecholamines by the adrenal medulla, such as norepinephrine and epinephrine. Another indicator of stress-related responses regulated by the sympathetic-adrenal-medullary axis is sAA, which is associated with both plasma norepinephrine and epinephrine changes and other indicators of sympathetic activity during physiological stress experiences (Baum, 1987; Chatterton et al., 1996; Rohleder et al., 2004; Nater et al., 2007; Nater and Rohleder, 2009; Thoma et al., 2012; Reyes del Paso et al., 2013). The second and slower response is the activation of the hypothalamic-pituitary-adrenal (HPA) axis, which leads to the release of glucocorticoids by the adrenal cortex (Wen, 1998; McEwen Harold and Milliken, 2000; Stroud et al., 2009; Ali and Nater, 2020). The HPA axis is the endocrine core of the stress system, which involves hypothalamic corticotropin-releasing hormone, pituitary corticotropin and adrenal CORT (De Kloet, 2004). Specifically, the HPA axis regulates the release of CORT, which is a key player in responding to environmental challenges (Heijnen et al., 2016). CORT levels are characterized by a circadian fluctuation where peak levels are reached about 30 min after waking and decline throughout the day, with a nadir at about midnight (Strahler et al., 2017; Jones and Gwenin, 2021). When a stressor is encountered, CORT levels gradually rise reaching a peak ∼20–30 min after the stressor onset and remaining elevated for ∼2 h (Chrousos, 2009). During this acute response to stress, these levels are self-regulated by a negative feedback loop (that is, from the adrenal gland to the hypothalamus and other brain regions such as the hippocampus and the frontal cortex) in order to shut the HPA axis down and return to a set homeostatic point (Lupien et al., 2007). When an acute exposure to a challenge is prolonged (e.g., an infection), CORT levels may remain elevated, although not at levels as high as the initial exposure. Prolonged exposure to stressors results in inappropriate basal activity such as higher CORT basal levels, and/or responsiveness such as disruptions of the acute HPA responses, which may cause physical, behavioral, and/or neuropsychiatric manifestations (Chrousos, 2009). Similar to CORT, sAA presents a distinct diurnal profile (Cozma et al., 2017). However, while CORT reaches peak values within 30 min of awakening, sAA levels drop sharply in the first 30 min of awakening and then steadily rise (Nater et al., 2007). It has been demonstrated that sAA secretion patterns, such as blunted levels at awakening, hyper- or hypo-secretions diurnally, and/or in reaction to acute stress, might serve as valuable indicators of the autonomic nervous system (ANS) dysregulations that typically occur in chronic stress-related pathologies (Nater et al., 2007; Ali and Nater, 2020).

There are many published manuscripts that have thoroughly and comprehensively reviewed the scientific literature on stress exposure, stress biomarkers, and neurocognitive function (Lupien et al., 2009; Epel et al., 2018). The current review is not intended to recapitulate the results from these prior reviews but instead focuses on whether changes in stress biomarkers could reflect a pathway by which prolonged exposure to PA, in the form of AE, influences neurocognitive function. As such, we provide here only a brief review of the well-documented science on stress biomarkers and neurocognitive function, and point the reader to other reviews for a more thorough and comprehensive summary of this vast field (Lupien et al., 2009; Epel et al., 2018).

Stress exposure, especially chronic exposure, adversely impacts cognition (Lupien et al., 2007; Juster et al., Lupien, 2010) and brain structure (McEwen and Gianaros, 2011). Using biological markers of stress exposure (i.e., CORT and sAA) and instruments that collect self-reported experiences to chronic stressors, research has demonstrated that chronic stress exposure negatively affects numerous cognitive processes including cognitive flexibility and working memory processes supported by the prefrontal cortex (PFC) (Arnsten, 1998; Arnsten, 2009), as well as memory processes regulated by the hippocampus (Luethi et al., 2009; McEwen and Gianaros, 2011). Decades of non-human animal research indicate that prolonged exposure to CORT is associated with memory impairment and with plastic remodeling of hippocampal circuitry (e.g., shortening of dendrites, loss of spine synapses, and suppression of neurogenesis in the dentate gyrus) (McEwen and Gianaros, 2011). These stressor-induced modifications in molecular and cellular processes are thought to be mirrored in humans that experience chronic stress and can be detected using neuroimaging techniques. For example, numerous studies have demonstrated that greater chronic stress exposure is associated with smaller hippocampal volumes (McEwen et al., 2007; McEwen and Gianaros, 2011). Furthermore, progressive increases in CORT levels have been shown to reduce performance on hippocampal-dependent memory tasks (i.e., tasks relying on episodic and relational memory) (Lupien et al., 1998; McEwen et al., 2007; McEwen and Gianaros, 2011) and executive functioning (MacLullich et al., 2005). Recent human studies have also found that chronic elevation of sAA levels is associated with cognitive impairment (Granger et al., 2007; Suh, 2021; Yamane et al., 2022). Acute and repeated stress exposure causes alterations in brain activity patterns viewed with PET and fMRI, and a reduction in the volume of brain regions, such as hippoampus and amygdala (McEwen et al., 2007). In sum, there is evidence from both human and non-human animal studies that suggests chronic stress exposure and elevated CORT and sAA concentrations are associated with impairments in cognitive function and brain morphology and function.

## 3 Effects of AE on stress biomarkers

**FIGURE 2:**
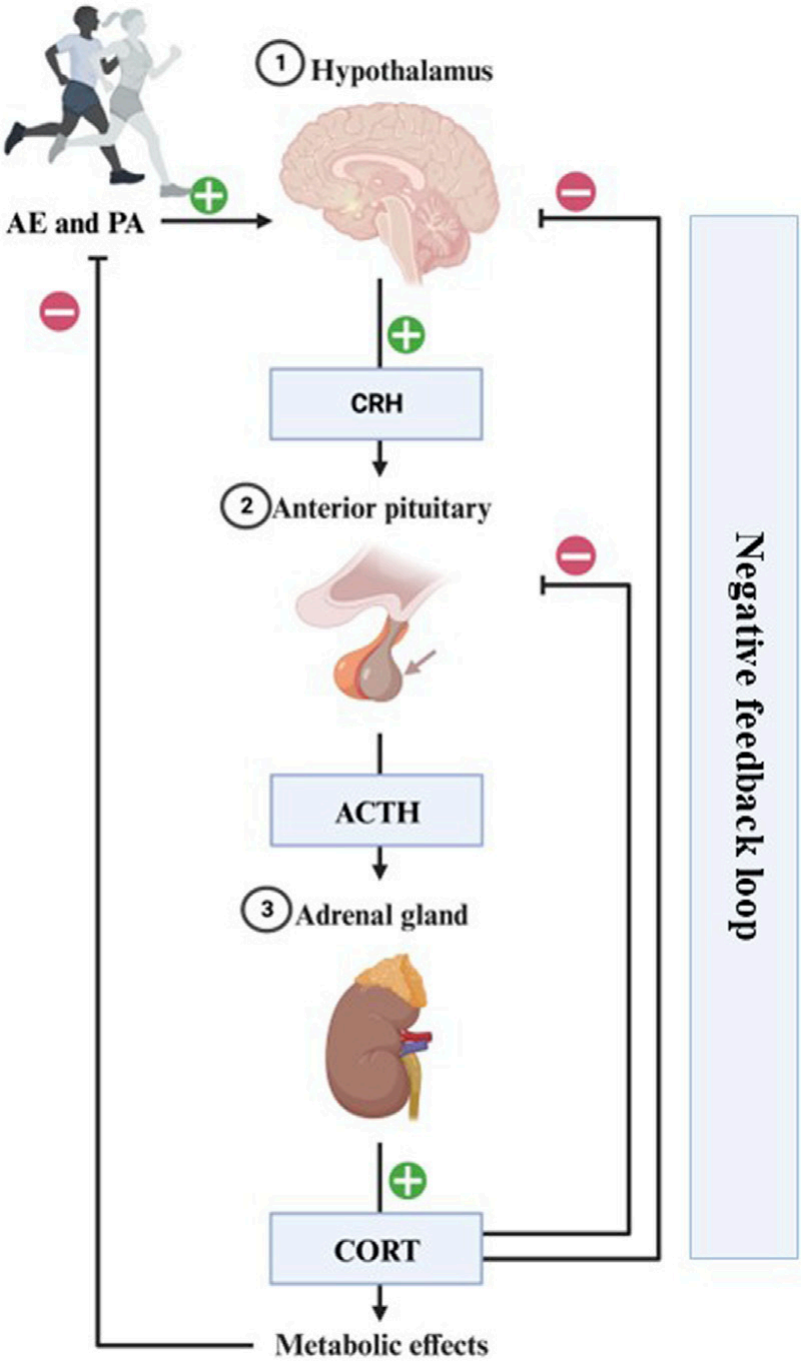
Representation of the physiological stress response to AE/PA. The physiological stress response starts with the hypothalamus secreting corticotrophin-releasing hormone (CRH), which travels to the anterior pituitary to include adrenocorticotropic hormone (ACTH) release into the general circulation. This hormone reaches the adrenal gland resulting in CORT release into the system. This has an inhibitory effect upon the hypothalamus and pituitary through medial PFC receptors, and also reduces amygdala overexcitability due to stress. Completion of this circuit restores homeostasis and is a sign of a healthy stress-response.

Physical and psychological stressors can activate the HPA axis and the ANS (Wood et al., 2018). The physiological stress response starts with the activation of the hypothalamus and pituitary gland, which secretes corticotrophin and adrenocorticotropic hormones, respectively. This last hormone reaches the adrenal cortex resulting in CORT release, which has an inhibitory effect upon the hypothalamus and pituitary through medial PFC receptors, and also reduces amygdala overexcitability due to stress (see Figure 2) (Hill et al., 2011; Heijnen et al., 2016).

In this regard, AE has been recognized as a type of stressor that can modify circulating levels of stress biomarkers (Heijnen et al., 2016). Although AE activates the body’s stress response through the HPA axis and ANS activation, these neural consequences might be different than those of negative life events because AE is accompanied by an increase in growth hormone and inactivation of CORT into cortisone (Hill et al., 2011). While single brief bouts of AE acutely elevate stress biomarker levels (Strahler et al., 2017), habitual engagement in AE might be associated with a faster recovery of the SNS and HPA axis in response to an alteration in baseline resting levels. This physiological change following prolonged engagement in AE may result in attenuated responses to the experience of a physical or psychological stressor (Strahler et al., 2017; Wood et al., 2018; Strahler and Wunsch, 2021).

Several explanations have been presented to describe the attenuating effect of prolonged exposure to exercise on stress biomarkers. For example, the “*cross-stressor-adaptation (CSA)*” hypothesis proposes that exercise can facilitate diverse adaptive physiological responses (i.e., cardiovascular tone or energy mobilization) affecting the HPA axis and the ANS (Sothmann, 2006). As an example, elevated catecholamine and glucocorticoid levels during PA and AE mobilize and replenish, respectively, the energy stores needed under physiological challenges (Heijnen et al., 2016). These biological adaptations contribute to reduced and optimized physiological reactions to other stressors (e.g., psychological or cognitive), resulting in an overall reduction in the secretion of stress biomarkers (Klaperski et al., 2014; Strahler et al., 2017). Previous studies have demonstrated that these modifications on stress pathways resulting from acute bouts of AE could persist for up to several days (Strahler et al., 2017). Taken together, higher levels of AE, PA, and fitness are associated with reduced reactivity and faster recovery of the HPA axis and ANS in response to stressors (Wunsch et al., 2019a). Thus, neuroendocrine and physiological adaptations to stressors may be optimized by improvements in physical fitness, minimizing the disruption of homeostasis (Sothmann et al., 1996). However, some potential effects of habitual exercise or fitness on physiological and neuroendocrine stress reactivity still remain unclear (Mücke et al., 2018). In the below sections, we will cover these stress system adaptations (i.e., HPA axis and ANS) related to acute and chronic exercise in greater detail.

### 3.1 AE and CORT

There are two main points that have been highlighted in the AE-CORT literature: i) there is substantial evidence that acute bouts of AE transiently increase CORT levels, but ii) promising–but methodologically weaker-evidence that chronic exposure to AE attenuates the CORT response to psychosocial stressors (Strahler et al., 2017).

There is a wealth of data demonstrating that acute bouts of AE transiently elevate CORT levels (Adsuara Cadegiani et al., 2020). This acute exercise-related CORT response seems to be higher when the volume or intensity of exercise is more elevated (Kraemer et al., 1995; Adsuara Cadegiani et al., 2020). Hence, the acute CORT response can be influenced by exercise intensity and duration, but also exercise training status (Hill et al., 2008). For instance, Hill et al. (2008) found that short (∼30 min) bouts of moderate-to-high-intensity AE (60% and 80% of maximal oxygen uptake) was effective at eliciting an acute CORT response because these intensities presented a challenge to physiological homeostasis (Hill et al., 2008). Elevations in salivary CORT have also been found after 30 min of high-intensity exercise when 4%–6% of the total training time was above 90% of maximum heart rate in healthy adults (Gottschall et al., 2020). Similar results have been found in athletes when the intensity was between 55% and 80% of the maximum individual work rate during a 30-min exercise test using a cycle ergometer (Hough et al., 2021). Taken together, these findings, and others, suggest that ∼30 min of moderate-to-vigorous exercise is sufficient to acutely elevate CORT levels.

Interestingly, a different CORT dynamic has been observed during longer bouts of exercise (>2 h). In this case, there is an initial increase in CORT after 20–30 min of exercise followed by a decrease in CORT (to pre-exercise or lower basal levels), and finally, a second increase in CORT after ∼2-h of exercise, with this second rise more pronounced in trained individuals (Viru and Viru, 2004). This biphasic increase in the pituitary-adrenocortical activity seems to be accompanied by a decreased sensitivity to CORT, which has been suggested as a possible adaptive mechanism to reduce an inflammatory reaction and protect the organism from exercise-induced muscle damage (Duclos et al., 2003; Viru and Viru, 2004). Similar increases in CORT have been found in marathon or ultra-marathon runners who show greater inactivation of CORT into cortisone than untrained individuals in resting conditions, thereby leading to more favorable muscle recovery (Duclos et al., 2003). Nonetheless, fewer controlled laboratory studies have examined the impact of prolonged (e.g., 2 h) exercise exposure on CORT and so less is known about the CORT dynamics and response.

There is also evidence that acute bouts of AE influences stress reactivity after ending the exercise exposure. For example, Zschucke et al. (2015) found that participants engaging in an acute 30-min AE intervention (at 60%–70% of maximal oxygen uptake) showed lower salivary CORT responses to a psychosocial stressor 90 min after the completion of the exercise relative to control participants not receiving exercise (Zschucke et al., 2015). Further, individuals with higher fitness levels showed greater blunting of the CORT response and greater hippocampal activation and decreased PFC activity during the psychological stressor (Zschucke et al., 2015). This suggests that even a brief exercise exposure could have extended effects on physiological responses to stress. Changes in stress biomarkers might also be related to the intensity of the exercise exposure. For example, Luger et al. (2010) found that treadmill exercise led to intensity-dependent elevations in plasma CORT concentrations (higher intensity led to higher concentrations), and this activation was inversely proportional to the level of physical training of the individuals (Luger et al., 2010). Similar results have been found showing that fitter individuals show attenuated SNS and HPA axis responses to a psychosocial stressor (Rimmele et al., 2009; Martikainen et al., 2013).

In contrast to the studies on acute exercise, the effect of more chronic exercise behaviors and interventions on CORT levels is more equivocal. Complicating this issue is lack of clarity regarding the amount of exercise exposure needed to be considered ‘chronic’ or ‘habitual’. Unfortunately, intervention studies that increase exposure to AE over long periods of time (e.g., 6 months) and examine basal levels of CORT or changes in CORT in response to a stressor remain relatively sparse and have methodological limitations that prevent definitive conclusions (Alghadir et al., 2015; Gothe et al., 2016; Beserra et al., 2018; Vrinceanu et al., 2019). For example, 3 sessions per week (60 min/session) of moderate-intensity AE training for 4-week (at 65%–75% of the maximum heart rate) increased salivary CORT in young adult men (N = 16) as measured by a single fasting sample taken mid-morning 24-h after training (Alghadir et al., 2015). In contrast, Vrinceanu et al. (2019) found that a 3-month dance/movement training, but not a 3-month AE training program, decreased salivary CORT measured by the cortisol awakening response (CAR) in healthy sedentary older adults (N = 12) (Vrinceanu et al., 2019). However, only the participants involved in the AE training improved cardiorespiratory fitness. In addition to these studies being limited by small sample sizes, the single assessment of salivary CORT (e.g., CAR) greatly limits interpretability.

Most studies examining the effect of regular exercise behaviors on the HPA axis emerge from cross-sectional work examining whether self-reported engagement in exercise or measurements of fitness or PA levels are associated with individual variation in patterns of CORT responses. Several studies have reported that higher cardiorespiratory fitness levels are associated with reduced physiological responses (including CORT reactivity) to acute AE bouts compared to lower-fit individuals when exercising at the same intensity (Viru et al., 2001; Viru and Viru, 2004; Hill et al., 2008; Klaperski et al., 2014; Strahler et al., 2017; Mücke et al., 2018). Yet, a systematic review of studies examining the influence of regular PA or fitness on stress reactivity as measured by the Trier Social Stress Test reported a mixed pattern with only 7 of 14 studies showing that CORT and heart rate variability were attenuated in adults engaging in greater amount of PA or who had higher fitness levels (Mücke et al., 2018). The authors highlighted the limitations of this largely cross-sectional literature. As an example, Wood et al. (2018) found that higher fitness levels were associated with lower CORT secretion in response to a psychological stressor in a non-athletic sample (Wood et al., 2018). Similar decreases in CORT reactivity to a psychological stressor were found in more physically active young adult women compared to low active women (Klaperski et al., 2014). Likewise et al. (2016) found evidence of blunted salivary CORT response to acute psychosocial stress in high- and moderate-fit males compared to less fit males (Strahler et al., 2017). Yet, other studies have failed to find any differences in CORT reactivity to stress as a function of cardiorespiratory fitness levels (Childs and de Wit, 2014; Jayasinghe et al., 2016; Strahler et al., 2017; Mücke et al., 2018).

One difference between the physiological response to exercise-stress and non-exercise stress is that exercise-stress is followed by an increased inactivation of CORT into cortisone, which seems to be a crucial mechanism to protect athletes against the deleterious effects of prolonged increased CORT secretion (Gouarné et al., 2005; Heijnen et al., 2016; Adsuara Cadegiani et al., 2020; Kraemer et al., 2020). This protective mechanism has been evidenced in triathletes, who presented a higher inactivation of CORT into cortisone than untrained individuals in resting conditions (Duclos et al., 2003). Different hypotheses have attempted to explain this paradoxical pattern, such as hypercortisolism during exercise and post-immediate exercise recovery accompanied by a decreased sensitivity to CORT. A systematic review suggested that chronic exercise increases basal CORT levels by decreasing glucocorticoid receptor sensitivity and this is followed by an enhanced novel stress-response of CORT (Chen et al., 2017). Along these lines, the *CSA* hypothesis postulates that favorable adaptations to AE that occur due to repeated exposure to exercise could be generalized to responses to other challenges in daily life (Sothmann, 2006). This hypothesis attempts to explain why individuals who are more physically fit have better CORT profiles and might show lower stress reactivity responses to stressors (Klaperski et al., 2014; Strahler et al., 2017; Wunsch et al., 2019b; Strahler and Wunsch, 2021).

In summary, a wealth of data indicates that acute exercise increases CORT while there are methodologically weaker and more inconsistent results from studies examining whether chronic exercise exposure has an attenuating effect on CORT reactivity in adult humans. There is evidence from cross-sectional studies that chronic exposure to exercise may exert a protective mechanism by increasing basal CORT levels due to decreased sensitivity of glucocorticoid receptors.

### 3.2 AE and sAA

Studies examining associations between AE and sAA are not as common as those studying CORT. In a narrative review, Koibuchi and Suki (2014) reported that exercise of different modalities (e.g., bicycle/cycle ergometer, treadmill, taekwondo, swimming, handcycle) consistently increased sAA activity and concentrations at exercise intensities ranging from 50% to 80% maximal oxygen uptake in young, healthy individuals (Koibuchi and Suzuki, 2014). Similarly, Mckune et al. (2014) found elevated sAA levels up to 2 h after two 60-min bouts of downhill running separated by 14 days (75% maximal oxygen uptake) in young un-trained males (N = 11) (McKune et al., 2014). Zschucke et al. (2015) found increases in sAA levels after a 30-min AE intervention (60%–70% maximal oxygen uptake) in a sample of endurance trained males (N = 20) (Zschucke et al., 2015). Similar to research on CORT, the effect of exercise on sAA are likely dependent on the intensity and duration parameters of the exercise. In support of this, Sariri et al. (2010) found an increase in sAA after short bouts of low-to-moderate intensity exercise (50%–75% maximal oxygen uptake) completed on a treadmill in a non-athletic male sample (N = 10) but the duration of the sAA activity level was positively correlated with exercise intensity (Siriri and Damirchi, 2010).

Similar to the work on CORT, it is likely that exercise-induced changes in sAA are modified by cardiorespiratory fitness levels. In support of this, Kunz et al. (2015) reported that higher fitness levels were associated with lower sAA baseline concentrations, but greater post-exercise increases in sAA concentration and secretion in fit cyclists (N = 17) (Kunz et al., 2015). Also replicating CORT results, the associations between exercise behaviors and psychosocial stress reactivity are muddy and largely inconclusive. In a sample of 302 male recruits to Swiss Army training, Wyss et al. (2016) found that higher cardiorespiratory fitness was associated with reduced sAA responses to acute psychosocial stress (Trier Social Stress Test) (Wyss et al., 2016). Another cross-sectional study found that engaging in regular exercise was associated with a faster sAA recovery after the Trier Social Stress Test (Strahler and Wunsch, 2021). Similar results were reported by Wunsch et al. (2019a), who found that acute and habitual exercise positively affects psychological stress reactivity in a sample of male adults (N = 84) (Wunsch et al., 2019b). In this study, active individuals who reported to be engaged in AE for at least 150 min/week during the prior 6 months were compared to low-active participants. Participants from both groups were randomized to either an acute exercise session using ergometer bicycling (70% of their individual maximum load in Watts/kg) or to a light stretching control group. The high active group showed reduced stress reactivity to the Trier Social Stress test and faster recovery for salivary CORT and sAA compared to the low active group (Wunsch et al., 2019a). In contrast, Zschucke et al. (2015) did not find associations between exercise levels and psychosocial stress-induced sAA in a sample of sedentary and highly trained (intensively trained in an endurance sport at least three times a week) young men (N = 40) (Zschucke et al., 2015). Also et al. (2017) did not find correlations between cardiorespiratory fitness and sAA reactivity in a sample of sedentary to low-active males (N = 115) (Strahler et al., 2017).

To summarize, acute exercise attenuates sAA reactivity similar to the effects found for CORT. However, whether chronic exercise in the context of controlled interventions impacts sAA levels remains an open question. Further, there is some evidence that exercise behaviors might modify sAA reactivity to psychosocial stressors, but the evidence is inconclusive and limited by mostly cross-sectional studies instead of laboratory-controlled manipulations of exercise behavior. Variation in study design, the tests used to induce psychosocial stress, and the sample investigated (e.g., un-trained or trained population) is also likely contributing to the heterogeneity of findings across studies.

## 4 AE effects on neurocognitive health

In contrast to the detrimental effects of chronic stress, engaging in exercise exerts beneficial effects on cognitive and brain outcomes (e.g., improves memory/cognition, and promotes structural and functional brain plasticity) (Hillman et al., 2008; Erickson et al., 2013; Stillman et al., 2016; Stillman et al., 2020). Many systematic reviews and meta-analyses have summarized this literature (Baker et al., 2010; C; Groot et al., 2016; Colcombe et al., 2003; Kelly et al., 2014; Northey et al., 2018). What remains to be clearly determined are the mechanisms by which chronic exercise influences neurocognitive health in humans (Stillman et al., 2016; Stillman et al., 2020).

There are multiple possible pathways explaining how exercise influences neurocognitive health. Animal models have shown that voluntary exercise affects neurogenesis and neuronal survival in the dentate gyrus subfield of the hippocampus (Kobilo et al., 2011; Biedermann et al., 2016; De Sousa Fernandes et al., 2020). Also, human and non-human studies have shown that exercise increases the expression of neurotrophic factors involved in learning and memory (Erickson et al., 2011; Leckie et al., 2014; Tari et al., 2019). AE may modulate central nervous system effects including increasing cerebral vascularization and blood flow, which might allow for increased nutrients and oxygenation (Rooks et al., 2010; Morland et al., 2017; Stillman et al., 2021), antioxidant production (Souza et al., 2022), and epigenetic changes (Liang et al., 2021). Further, neuroimaging studies in humans demonstrate that AE and PA are positively associated with volume of the hippocampus and PFC in healthy and clinical samples (Erickson et al., 2011; Erickson et al., 2014; Maass et al., 2015; Morris et al., 2017). As described above, the hippocampus and the PFC are involved in, and affected by, the HPA axis (Herman et al., 2005) indicating overlap in the brain regions affected by exercise and the HPA axis, but with effects in opposing directions.

Because of the associations between AE and HPA-axis biomarkers, HPA-axis biomarkers and cognitive function, and between AE and cognitive function, it has been speculated that exercise-induced modifications in HPA-activity and stress-reactivity could be one mechanism by which the cognitive effects of exercise are realized.

## 5 AE, stress biomarkers, and neurocognitive health

In this section, we describe the evidence that examines whether AE-induced or PA-induced changes in stress physiology could be a plausible biological pathway by which exercise influences cognition. For instance, Gothe et al. (2016) found that an 8-week yoga intervention in sedentary older adults (N = 118) improved working memory performance and this improvement was mediated by an attenuated CORT response (Gothe et al., 2016). This study, although not an intervention of AE, hints at a possibility that changes in stress pathways could be a mechanism for AE-induced cognitive benefits. However, mindfulness activities are an important component of yoga, and it is unclear whether the mindfulness component of yoga was driving the downregulating effect on the ANS and the HPA axis in response to stress or whether this was driven by the PA component of yoga. In this regard, a RCT comparing a 90-min class of African dance or Hatha yoga in healthy college students (N = 69) showed that both interventions reduced perceived stress (West et al., 2004). However, African dance increased CORT levels and these changes were positively correlated with positive affect, whereas yoga decreased CORT levels, which was negatively correlated with positive affect (West et al., 2004). These results suggest that even when these interventions have positive psychological effects, they may differ in the physiological stress pathways involved.

The few studies that have examined the effects of AE interventions on cognitive function and peripheral measures of HPA and SNS activity show mixed results (Kraemer et al., 2006; Baker et al., 2010; Hötting et al., 2016; Drogos et al., 2019; Wang et al., 2019; Suh, 2021). Baker and others (2010) measured the effects of a 6-month AE intervention on cognition and plasma CORT in 33 older adults with mild cognitive impairment (Baker et al., 2010). Plasma CORT was measured from a single fasting blood draw before and following the intervention. The AE group showed reductions in CORT as well as improvements in executive functioning compared to controls after the 6-month intervention. Interestingly, women showed stronger effects for both CORT and executive function improvements suggesting the possibility of sex-specific effects (Baker et al., 2010). However, the small sample size precluded the use of statistical moderation or mediation approaches to examine this possibility. The only study to date examining the effect of AE training on CORT and cognitive function in cognitively normal older adults came to a different conclusion regarding the role of CORT in AE-related cognitive changes. Before and after a 6-month AE intervention in 32 healthy but sedentary older adults, Drogos et al. (2019) measured the CORT CAR and daily area under the curve, along with cognitive functioning assessed through a broad neuropsychological battery measuring several cognitive domains (i.e., processing speed, executive function, verbal memory, fluency, and attention) and perceived stress through a questionnaire. CORT was measured via saliva samples collected before and after the intervention at waking, as well as 15-, 30-, and 45-min post-waking and 3-, 6-, 9-, and 12-h post-waking (a total of 8 samples collected at each time point) (Drogos et al., 2019). They found that CAR increased following the intervention, but there were no significant changes in daily AUC (Drogos et al., 2019). No associations were found between changes in CAR nor daily AUC and cognitive changes. Both the Baker et al. (2010) and Drogos et al. (2019) studies were likely insufficiently powered to test for mediating effects of exercise-induced CORT changes on cognitive outcomes (Baker et al., 2010; Drogos et al., 2019). Further, both studies used different measures of CORT (saliva and plasma, respectively) and different collection frequencies to estimate daily AUC. This is a critical limitation as diurnal variation can significantly influence CORT values obtained at single points, and multilevel models suggest that measures taken across at least 3 days are needed to reliably estimate average CORT activity (Kraemer et al., 2006). Furthermore, measurement of salivary CORT seems to more accurately determine CORT concentrations than measurements of plasma CORT (Blair et al., 2017; El-Farhan et al., 2017). Thus, using different sampling methods adds more variability and makes comparisons between assays difficult. Finally, there was no control group in the Drogos et al. (2019) study, nor were overall cognitive changes reported (Drogos et al., 2019). This limitation makes it impossible to determine whether unique characteristics of the sample or aspects of the training program were responsible for the observed pattern of effects.

Could the effects of acute exercise on cognitive outcomes be mediated by changes in stress biomarkers? Hötting et al. (2016) assessed the effects of a single bout of physical exercise on memory consolidation and the possible underlying neuroendocrinological mechanisms in 82 young and healthy adults (Hötting et al., 2016). Participants encoded a list of words before a 30-min bout of exercise (either high- or low-intensity), and salivary CORT was measured at baseline, after learning, and after the exercise. They found that the high-intensity exercise group (∼80% heart rate max), showed an increase in CORT and exhibited better memory consolidation compared to the control group (24 h after the learning process), but no differences in recalled words (N = 81) (Hötting et al., 2016). Moreover, they did not find a significant relationship between memory scores and salivary CORT levels, suggesting that alterations in CORT were unlikely mediating exercise-induced changes in cognition (Hötting et al., 2016; Ponce et al., 2019; Wang et al., 2019).

We found only two studies that have included sAA and cognition measurements in the context of PA (Wunsch et al., 2019a; Suh, 2021). One study aimed to investigate the associations between PA, objectively measured by accelerometry, and sAA and salivary CORT on cognitive function in 98 hospitalized older adults with mild cognitive impairment (Suh, 2021). They found that lower salivary CORT levels, but higher sAA levels and higher PA levels (i.e., volume and intensity) were associated with better cognitive function as measured by the Mini-mental State Examination and executive function tests (Suh, 2021). These results concur with those reported by Wunsch et at. (2019b), who found that PA buffered the negative effects of stress on cognitive performance in a sample of 44 preadolescent children (Wunsch et al., 2019a). In this study, regular PA was assessed for seven consecutive days using accelerometers and saliva CORT and sAA samples were collected at six time-points after a psychosocial stressor (0, 13, 23, 50, 60, and 80 min with reference to the end of the resting period). Interestingly, those physically active children who exhibited lower levels of sAA or higher salivary CORT after the stressful task exhibited superior working memory performance (Wunsch et al., 2019b). These findings suggest that PA may exert stress-buffering effects on cognitive performance in children (Wunsch et al., 2019a). Importantly, none of these studies were able to conclude that exercise is exerting positive effects on cognitive functioning through changes in stress biomarkers.

There are three main sources of heterogeneity in the methods and results across studies that have examined links between exercise, cognition, and stress biomarkers. First, different collection methods, and intervals of collection for stress biomarkers are used across studies. This variation makes it difficult to understand the outcomes and interpretations of the CORT, sAA, or other results. Second, there is significant heterogeneity in the length and types of exercise interventions employed without clearly defined parameters. Since there is evidence that this impacts CORT and sAA (as well as brain and cognition), this heterogeneity is likely contributing to the various patterns of results. Third, studies were conducted in highly diverse samples, ranging from children to older adults and from healthy to patient groups. This makes it difficult to understand whether stress pathways are a possible mechanism for exercise-related changes in cognition as the sample characteristics could conflate the outcomes. For instance, the secretion of CORT and sAA varies across the lifespan (i.e., attenuated slopes and increases in daily levels of CORT, as well as increases in sAA levels in older adulthood).

## 6 Discussion

The present review summarized the results from studies examining associations and effects of AE and PA with markers of peripheral pathways that respond to stress, particularly CORT and sAA, and how these patterns might relate to neurocognitive changes associated with AE. We, and others, have speculated that the neurocognitive benefits of exercise might be mediated by exercise-induced changes in the activation of stress pathways (Stillman et al., 2016). Our conclusion is that despite the intuitive appeal of this hypothesis, there is little data currently available to support it. The lack of supporting data is driven by few studies examining the hypothesis and considerable heterogeneity in methodology and sample characteristics across the studies that have tested this hypothesis.

Whereas psychological stress impairs cognitive functions, exercise has been shown to exert physiological, psychological, and cognitive benefits. Stress biomarkers such as CORT or sAA have been discussed as possible neuroendocrinological explanatory factors of exercise induced cognitive changes (e.g., improved memory). However, there is an absence of studies that have examined biomarkers of HPA axis or ANS activities as proxies for the mechanisms underlying the neurocognitive benefits of AE training. In fact, only a few AE interventions to date have measured both stress biomarkers (CORT and/or sAA) and cognitive outcomes in the same study. However, these studies have been focused on acute effects of AE interventions. Because of the wealth of controlled laboratory data of acute exercise effects on stress biomarkers, it can be hypothesized that the acute exercise-induced benefits to cognition are mediated through this pathway. But the lack of long-term well-controlled laboratory exercise experiments or randomized exercise interventions on changes in stress biomarkers limits support for the hypothesis. Most of the literature in this area emerges from cross-sectional studies or are of short duration. In contrast, there is a wealth of literature about chronic stressors and stress biomarkers on neurocognitive function and the benefits of long-term exercise behaviors. But, as described above, studies that have linked exercise-induced changes in stress pathways to neurocognitive changes are scarce. More longitudinal and RCT studies are needed to examine the impact of long-term exercise or dose of exercise on CORT and sAA, and whether changes in CORT and/or sAA mediate exercise-induced neurocognitive benefits.

On the other hand, it has been suggested that dysregulation of stress reactivity may represent a mechanism by which psychological stress contributes to the development of future health and disease outcomes (e.g., cognitive impairment, mental health outcomes). Similarly, early life exposure to stressful events can have protracted effects on the brain and cognition, which might be mitigated by engagement in exercise behaviors (Donofry et al., 2021). Following these lines, a protective effect over allostatic load has been attributed to exercise, where AE seems to improve stress reactivity leading to a better stability through stressful events that harm body homeostasis. Although acute exercise seems to influence stress reactivity (e.g., sAA and CORT reactivity), the evidence is limited by mostly cross-sectional studies instead of laboratory-controlled exercise interventions. Therefore, whether chronic exercise in the context of controlled manipulations show modifications in sAA or CORT levels or reactivity remains an open question.

In conclusion, we know that while acute and chronic exercise promotes cognitive and brain health, acute stress is detrimental and chronic stress exposure is closely linked to many diseases and psychological conditions like anxiety or depression. The benefits of acute and chronic exercise on cognitive function and stress reactivity have been extensively covered in the literature; however, no studies have examined the long-term effects of exercise on stress pathways as a mediator for cognitive improvements. Based on this, our conclusion from this review is that a greater number of well-controlled studies are needed to adequately test the hypothesis that exercise-induced changes in the activation of stress pathways might mediate neurocognitive benefits of exercise. Furthermore, developing novel models that focus on different populations, such as those with primary adrenal insufficiency (Addison’s disease), may aid in understanding the effects of exercise on neurocognitive metrics in individuals with normal cortisol responses *versus* those with clamped “normal” cortisol.
